# Contributions of Positive Psychology in Self-Regulated Learning: A Study With Brazilian Undergraduate Students

**DOI:** 10.3389/fpsyg.2019.02980

**Published:** 2020-01-28

**Authors:** Vanessa Kaiser, Caroline Tozzi Reppold, Claudio Simon Hutz, Leandro S. Almeida

**Affiliations:** ^1^Psychological Assessment Research Laboratory, Federal University of Health Sciences of Porto Alegre, Porto Alegre, Brazil; ^2^Department of Psychology, Federal University of Rio Grande do Sul, Porto Alegre, Brazil; ^3^Department of Educational Psychology and Special Education, University of Minho, Braga, Portugal

**Keywords:** self-regulated learning, positive psychology, academic adaptation, self-efficacy, positive affects

## Abstract

Self-regulated learning (SRL) is an important factor for academic success. The present study aimed to investigate the relationship between constructs typical of positive psychology (PP; self-esteem, self-efficacy, affects, life satisfaction, optimism, and hope) and SRL while indicating the explained variance of these constructs in an SRL model. The study comprised 1,046 undergraduate students from 63 public higher education institutions from all demographic regions of Brazil. Significant correlations (*p* < 0.01) were found between SRL and all PP variables. Moderate correlations were found with self-efficacy and positive affects. The linear regression analysis indicated that one model explains 41.9% of the variance in SRL. The data are discussed based on the potential of PP and SRL interventions to improve academic performance and student adaptation to higher education environments.

## Introduction

Self-regulated learning (SRL) is defined as the dynamic use of resources by an individual to organize cognitive processes and manage internal and external elements aiming to reach a goal (Schunk and Zimmerman, 2012; Cueli et al., 2017). It is a construct to be understood as a continuum instead of as a dichotomy (such as presence versus absence), which indicates a more autonomous or controlled behavior in learning activities compared to peers (Wijnia et al., 2011; Becker, 2013; Gandomkar and Sandars, 2018). In applied terms, studies have shown that SRL predicts academic and social fitting levels of an individual, with self-regulated students characterized as more resolute, strategic, and persistent during learning (Preto and Moreira, 2012; Iyama and Maeda, 2018).

**FIGURE 1:**
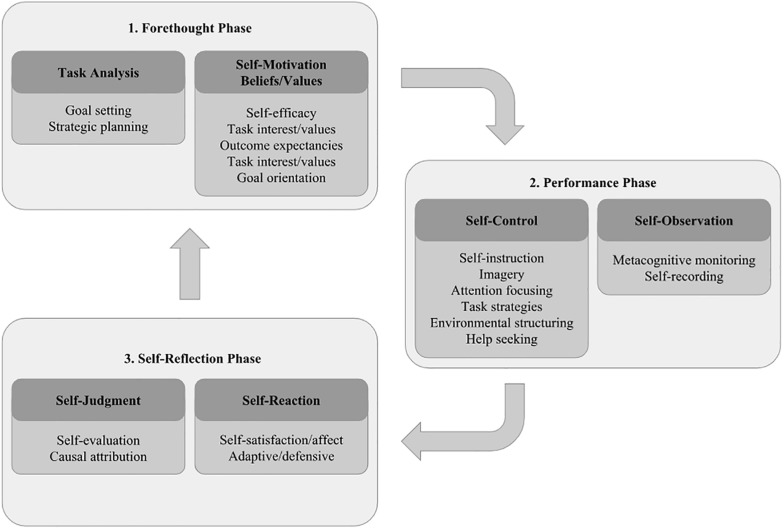
Cyclical model of self-regulated learning. Source: adapted from Zimmerman (2013).

Academic self-regulation involves the following phases: planning (establishing goals and time assignment), execution (selection and use of strategies; regulating time and effort), and evaluation (reflection on task fulfillment; comparison between intended and reached goals) (Zimmerman, 2000). According to the cyclical model proposed by Zimmerman (2013) to understand the construct, shown in Figure 1, each step involves specific processes.

In this viewpoint, the learning self-regulation process is closely associated with thoughts, feelings, and actions developed and guided by the student to carry out pre-established goals by systematically using cognitive, metacognitive, motivational, and behavioral strategies (Zimmerman, 2000, 2013). Zimmerman (2008) empirically identified 14 SRL strategies employed during the SRL phases and classified them into three categories: (1) *Metacognitive strategies* – include setting goals and planning, organization, and transformation, seeking information, and rehearsal and memorization; (2) *motivational strategies* – include self-evaluation and self-consequating; and (3) *behavioral strategies* – include environmental structuring, record keeping and monitoring, reviewing texts/notes/tests and seeking assistance from peers/instructors/other people holding knowledge. More recently, other actors have shown that the development of these self-regulation strategies is key for the school success of children (Arias and Díaz, 2010), adolescents (Bempechat et al., 2018), and adults (Wibrowski et al., 2017) as they increase the control by students over their learning processes.

However, SRL is multifactorial (de Sousa et al., 2013). Thus, personal beliefs of students regarding their capacities and performance (such as self-image, self-efficacy, and realization expectations) are also variables to be taken into account in the self-regulation process. Almeida (2007) indicated that academic adaptation and dedication of students to schoolwork are strongly associated with success expectations and failure experiences. The belief in self-efficacy has stood out in exploratory studies as one of the most important predictive variables in the learning process (Azzi and Polydoro, 2010; Demirören et al., 2016). This evidence assumes other constructs typically approached by positive psychology (PP), in theory related to self-efficacy, may predict the SRL of undergraduate students, which would lay scientific bases for future preventative interventions focused on implementing positive attributes in the academic context so as to enhance school performance. Indeed, seeking scientific evidence that support psychological practices is one of the most reinforced premises of PP by Seligman and Csikszentmihalyi (2000) toward a psychology that helps people become happier, more engaged and more fulfilled in their actions.

Studies developed under the viewpoint of positive education have indicated the relation between positive constructs and SRL. One such study is the meta-analysis carried out by Sitzmann and Ely (2011) on SRL among adults, which reveals that the variables with the highest correlations and effects on learning are goal level (*p* = 0.44, *k* = 24, *N* = 3.565), self-efficacy (*p* = 0.35, *k* = 60, *N* = 25.798), effort (*p* = 0.28, *k* = 67, *N* = 8.569), and persistence (*p* = 0.27, *k* = 30, *N* = 6.979). The importance of affects in the adaptation to higher education life is pointed out in international studies that indicate emotions are directly associated with student motivation for learning (Ahmed et al., 2013; Mega et al., 2014; Webster and Hadwin, 2015). However, few studies have investigated positive health indicators and adaptation while relating them to SRL. The present study aimed to investigate SRL among Brazilian undergraduate students of different majors while relating this construct to sociodemographic variables and several positive constructs (self-esteem, self-efficacy, affects, life satisfaction, optimism, and hope). The predictive value of such constructs in an explanatory model of SRL in the higher education context was also investigated.

## Materials and Methods

### Criteria for Inclusion in the Sample

The study comprised undergraduate students of public (state or federal) higher education institutions in Brazil. Students from private institutions were excluded. Overall, 1,240 undergraduate students took part in the study, of whom 1,046 were included based on the eligibility criteria.

### Instruments

The following instruments were employed in data collection:

#### Sociodemographic Questionnaire

Questionnaire containing relevant information to the sample evaluated, including age, gender, course, institution, average grade over the previous semester, school shift, whether they intended to continue their studies, whether they worked, and the number of hours of work.

#### Rosenberg Self-Esteem Scale (Hutz et al., 2014)

Instrument comprising ten items answered through a Likert scale to assess overall self-esteem. The original scale has unifactorial structure, which was also found in validation studies of the scale adapted to Brazil. It has adequate levels of precision (Cronbach α = 0.90) and national validity evidence based on its internal structure, external criteria, and normalization structure for the Brazilian population.

#### Overall Self-Efficacy Scale (Pacico et al., 2014)

Composed of 20 items (14 positive and six negative) in which individuals choose on a Likert scale how much the sentence describes them. It has unidimensional structure with evidence of validity and adequate precision (α of 0.89).

#### Optimism Evaluation Test (LOT-R) (Bastianello and Pacico, 2014)

Self-report scale to evaluate dispositional optimism comprising ten items divided into three statements on optimism, three on pessimism, and four filter items whose scores are not taken into account. Respondents answer questions indicating how much they agree with the item using a five-point Likert scale. The instrument is a reduced and revised version of the Life Orientation Test – LOT (Scheier and Carver, 1985) that was adapted, validated, and normalized for Brazil and that has unidimensional structure, as the original instrument, and internal consistency of 0.80.

#### Positive and Negative Affects Schedule (PANAS) (Zanon and Hutz, 2014)

The instrument assessed the experience of positive and negative affects, emotional components of subjective well-being. It comprises a list of 20 words expressing emotions at ten items for each type of affect. Respondents use a five-point Likert scale to assess how much they felt the emotions described over recent weeks. The PANAS is the most widely employed instrument worldwide to assess affects and studies have shown its adequacy in the Brazilian context in terms of precision and validity evidence.

#### Life Satisfaction Scale (LSS)

Created by Diener et al. (1985), the Brazilian version of the scale was adapted and validated by Zanon et al. (2014). It comprises five self-report items whose contents assess the level of satisfaction of individuals with their life conditions. The answers are given using a seven-point Likert scale. Studies have shown that the scale comprises a single factor and has high internal consistency (α = 0.87) and high test-retest reliability (*r* = 0.82). The instrument has several pieces of evidence of internal structure validity and data on Brazilian normalization.

#### Cognitive Hope Scale (Pacico and Bastianello, 2014)

Composed of 21 items that measure altruistic hope and self-centered hope. The instrument comprises two columns, each representing a subscale: desire (how much you want something) and expectation (how much you believe that will happen). This instrument was created based on The Hope Index by Staats (1989) and its Brazilian version features five additional items compared to the original according the prior studies on construct content evidence in the Brazilian context. It has evidence of internal structure validity with Cronbach α coefficients of 0.86 for self-centered hope and 0.80 for altruistic hope, in addition to normalization data for Brazilian samples.

#### Scale of Self-Efficacy in Higher Education (Polydoro and Guerreiro-Casanova, 2010)

Comprising 34 items, it is answered through a ten-point Likert scale ranging from “little” to “a lot.” The scale assesses three self-efficacy dimensions in higher education, namely: (a) academic (confidence in the capacity to learn and apply the content), (b) education regulation (confidence in the capacity of setting goals and self-regulating actions), and (c) social interaction (confidence in the capacity of relating with colleagues and professors). The scale has normalization and validity evidence in Brazil, with adequate internal structure and α-values between 0.86 and 0.90 in the three dimensions.

#### Scale of Study Competency Evaluation (ACE) (Almeida et al., in press)

The scale aims to assess the study methods and SRL of undergraduate students. It was created based on the SRL Theory – particularly, on the Categories of SRL Strategies by Zimmerman (2002), which indicate initiatives by students themselves toward self-evaluation, organization and transformation, goal and planning, information seeking, record maintenance and monitoring, environment organization, self-consequences, social support seeking, record review, and model observation. The scale comprises 59 items answered through a five-point Likert scale.

### Procedures

The present study employed a cross-sectional design and its data collection was carried out at a single moment using an online questionnaire created using Google Forms. The research was publicized in two ways: (1) By e-mail, contacting all Brazilian public universities (course supervisors, provost’s office of teaching, and provost’s office of undergraduate studies), who forwarded the link of the research to students of their institutions and (2) through social networks.

The project was approved by the Research Ethics Committee of the Federal University of Health Sciences of Porto Alegre (CAAE 52105315.7.0000.5345/opinion no. 1,625,066) and met all ethical criteria of human research. The eligible population was invited to voluntarily take part in the study and had anonymity guaranteed.

### Data Analysis

First, descriptive analyses of the sample were performed. The statistical tests were chosen taking into account the Central Limit Theorem. The significance of differences among groups derived from sociodemographic information regarding SRL scores was verified using ANOVA and the intergroup differences were investigated by Tukey’s honestly significant difference test. Scores from PP instruments and SRL scores were correlated using the Pearson coefficient parametric test. In order to investigate the explanatory model of SRL, multiple linear regression analysis was used, being SRL the dependent variable and those from PP, the independent variables. Multiple linear regression enabled building models to verify the influence of positive attributes in explaining SRL, of which the most parsimonious was chosen. Variables were selected using the Stepway method.

The database was initially exported from Google Forms into the software Excel and then transformed to the software Statistical Package for Social Sciences – SPSS version 23.0, where the statistical analyses were performed considering significance level of 0.05.

## Results

The study comprised 1,046 undergraduate students of 63 public (state or federal) higher education institutions in Brazil. Of those, most (66.9%) were female. Participant age ranged from 18 to 65 years old with an average of 24,16 (SP = 6.54). Table 1 presents the data regarding sample characterization.

**TABLE 1:** Characterization of the sample of Brazilian undergraduate students.

Characteristic	*N*	%
**Gender**		
Female	700	66.9
Male	337	32.2
Others	9	0.9
**Field of major**		
Agrarian Sciences	116	11.1
Biological and Healthcare Sciences	306	29.4
Exact Sciences	267	25.6
Human and Social Sciences	353	33.9
**School shift**		
Morning	128	12.2
Afternoon	45	4.3
Evening	271	25.9
Mixed	602	57.6
**Mean grade in the previous semester**		
A (9 to 10)	132	17.3
B (8 to 8.9)	295	38.6
C (6 to 7.9)	294	38.4
D (below 5.9)	44	5.8
**Frequency of professional activity**		
Alternate shifts or no fixed hours	193	43.1
Part time	156	34.8
Full time	99	22.1
**Weekly work hours**		
20 or less	263	59.1
Over 20	90	20.2
Over 40	92	20.7
First graduation	866	82.8
Intends to conclude current graduation	1.011	96.7
Works	463	44.3

Initially, the categorical variables resulting from the sociodemographic questionnaire were compared among groups to test for possible differences associated with SRL. The results are presented in Table 2.

**TABLE 2:** Comparison among sociodemographic groups in self-regulated learning.

	*N*	Mean	*SD*	*F*_(gl)_	*p*
**Demographic region**				***F*_(__4__;__1_,_040__)_ = 2.75**	**0.03**

Northeast	114	226.2^a^	28.9		
South	364	222.4^ab^	27.5		
North	17	219.3^ab^	41.3		
Center-West	28	219.8^ab^	36.4		
Southeast	522	217.7^b^	29		

**Major field**				***F*_(__3__;__1_,_038__)_ = 3.86**	**0.01**

Biological and Healthcare Sciences	306	223.29^a^	28.4		
Agrarian Sciences	116	223.61^ab^	28.2		
Exact Sciences	267	220.56^ab^	27.4		
Human and Social Sciences	353	216.3^b^	30.6		

**Intends to conclude graduation**				***F*_(__1__;__1_,_044__)_ = 7.02**	**0.01**

Yes	1.011	220.76	28.6		
No	35	207.57	37.1		

**Academic performance**				***F*_(__3__;__761__)_ = 4.25**	**0.01**

A (9 to 10)	132	222.36^a^	28		
B (8 to 8.9)	295	220.44^ab^	28.2		
C (6 to 7.9)	294	214.53^b^	29.2		
D (below 5.9)	44	210.52^ab^	24.6		

*Means followed by the same letters do not differ according to Tukey’s test.*

The correlations obtained between SRL and the self-esteem, self-efficacy, affects, life satisfaction, optimism, and hope are described in Table 3.

**TABLE 3:** Correlation between self-regulated learning and positive psychology constructs.

	Self-regulated learning
	Pearson correlation
Academic self-efficacy	0.52^∗^
Self-efficacy in regulating studies	0.52^∗^
Self-efficacy in social interaction	0.48^∗^
Self-efficacy in pro-active actions	0.57^∗^
Self-efficacy in academic management	0.59^∗^
Life satisfaction	0.31^∗^
Positive affects	0.45^∗^
Negative affects	−0.16^∗^
Self-esteem	0.31^∗^
Optimism	0.27^∗^
Self-centered hope	0.23^∗^
Altruistic hope	0.16^∗^
Overall self-efficacy	0.46^∗^

*^∗^*p* < 0.01.*

Finally, the scores from the PP instruments were plugged into multiple linear regression models to study their effects on explaining SRL. Seven models were created using multiple linear regression, the last of which being the most parsimonious one. The variables included in these analyses were self-efficacy in academic management, self-efficacy in pro-active actions, positive affects, negative affects, academic self-efficacy, self-efficacy in social interaction, and overall self-efficacy. The model construction steps with the variables included and the explanation power are presented in Table 4.

**TABLE 4:** Multiple linear regression models of self-regulated learning.

Level	Variables included in the model	*R*^2^_adj_
1	Self-efficacy in academic management	0.34
2	Self-efficacy in pro-active actions	0.40
3	Positive affects	0.41
4	Negative affects	0.41
5	Self-efficacy in social interaction	0.42
6	Overall self-efficacy	0.42

The final model obtained was significant (*F*[6.1039] = 126.48, *p* < 0.001) and explained 41.9% of the variance in SRL. Table 5 presents the most significant variables for the final model and their regression coefficients to explain SRL.

**TABLE 5:** Multiple linear regression analysis for the positive psychology constructs.

Independent variables	Self-regulated learning (dependent variable)				
	Non-standardized coefficients		Standardized coefficients		*p*-value
	*B*	Standard error	β	*T*	
Constant	109.82	5.7		19.28	0.01
Self-efficacy in academic management	5.38	0.59	0.31	9.04	0.01
Self-efficacy in pro-active actions	3.76	0.60	0.24	6.29	0.01
Raw score of positive affects	0.37	0.12	0.11	3.11	0.01
Raw score of negative affects	0.37	0.08	0.12	4.48	0.01
Self-efficacy in social interaction	1.27	0.55	0.08	2.29	0.03
Overall self-efficacy scale	0.16	0.07	0.08	2.24	0.03

As shown in Table 5, the variable of self-efficacy in academic management is the one with the greatest power in the model, followed by self-efficacy in pro-active actions. The variables with the least power in the model are positive affects and overall self-efficacy.

## Discussion

The present study investigated the SRL of Brazilian undergraduate students under the viewpoint of the contributions of PP. The study specifically assessed (a) possible differences in self-regulation determined by sociodemographic characteristics or by the context of the student, (b) the relationships obtained between SRL and self-esteem, self-efficacy, affects, life satisfaction, optimism, and hope, and (c) the predictive value of the positive variables on SRL.

The results indicated differences in the mean SRL score when taking into account the field of study of the course, the grades achieved by the students, and student intention to conclude the current undergraduate course. The literature carries contrasting data regarding differences in the self-regulation of students of different fields. While Ribeiro and Silva (2007) reported no distinctions, Rosário et al. (2000), when working with a sample of students of the final year of high school split according to the undergraduate courses they planned to attend, reveals differences that match those of the present study. Students interested in scientific-natural areas (biology and healthcare) had higher self-regulation scores, whereas those in economic-social or humanities fields had lower SRL.

As expected, students with better school performance (grades) and those most motivated to conclude the undergraduate course had higher SRL compared to their peers. That can be explained by the motivational beliefs that make up the cyclical model proposed by Zimmerman (2013) and corroborate the study by Muwonge et al. (2017), which suggests that students with more motivational beliefs related to academic tasks and their efficacy use more metacognitive strategies to fulfill their assignments, which results in greater SRL capacity. As for academic performance in particular, the data found and the literature suggest SRL is related to academic adaptation. That highlights the importance of higher education institutions to provide an environment that promotes SRL strategies since low SRL levels are associated with lower student motivation, with increased intention to drop out, especially in the 1st year, and higher levels or procrastination (Zoltowski et al., 2016; Correia and Moura Júnior, 2017; Grunschel et al., 2018). Taking into account the positive attributes investigated, the results indicate all PP constructs assessed in the present study were significantly correlated with SRL, matching the hypothesis by the authors. The constructs with the highest correlations with SRL were self-efficacy and positive affects. Indeed, the Brazilian literature already features studies that have associated SRL with overall self-efficacy or academic self-efficacy (Azzi and Polydoro, 2010; Demirören et al., 2016). It is noteworthy that self-efficacy is part of the cyclical model proposed by Zimmerman (2013) to understand SRL, being featured in the first phase of the model (anticipation) and on the scope of motivational strategies. However, the results of the other positive variables are more incipient and refer exclusively to foreign researches. Internationally, the results on the established relation between SRL and affects found in this study match the reports by Mega et al. (2014). That research reported that research affects were positively related with performance self-assessment and metacognitive reflection during the study and that both positive (β = −0.53) and negative (β = −0.25) affects influenced student motivation. Notably, positive affects increased student belief in their intelligence and self-efficacy, which impacted the use of SRL strategies and academic performance. In addition, studies in the field of education have highlighted the importance of affects as predictors of emotional and educational adjustment and the attachment to the university (Farzaneh et al., 2016). Evidence exists from young-adult samples of significant positive correlations of this construct with life satisfaction (Ruvalcaba-Romero et al., 2017), extraversion (Noronha et al., 2015), intrinsic motivation (Bye et al., 2007), and flexible/creative thought and decision-making in complex environments (Baars et al., 2017), which are important aspects in the academic environment. The low magnitude found between SRL and self-esteem, optimism, and hope – constructs often considered in intervention plans developed in the higher education context – stands out.

The multiple linear regression indicated that self-efficacy and affects explained 41.9% of the variance in SRL in the sample investigated. These results are pertinent in face of the importance of self-efficacy in the model created by Zimmerman (2013) and highlight the relevance of taking such constructs into account when planning interventions to be carried out in higher education institutions so as to enhance student SRL. This is especially important in the current historic context of public higher education in Brazil. Since 2003, the country has undergone an extensive higher education reform with public policies aimed at broadening the access to free public higher education, with a higher number of enrollments in undergraduate courses, which provides access to higher education to social segments that have been historically excluded from it due to race and socioeconomic status) (Zago et al., 2016). However, the current challenge is to reduce drop out rates and create strategies to maintain those students in class in a healthy way. Interventions based on the precepts of PP that seek to increase SRL capacity may be beneficial to those students and to higher education as a whole as long as they have empirical bases on studies showing the relevance of the constructs to be taken into account in their execution.

In this viewpoint, the paper highlights the predictive value of self-efficacy and of affects on SRL. It must be considered, however, that future studies using longitudinal designs may contribute to understanding the causal relations established among those variables. One strong aspect of the study is that the variables considered to be predictive of SRL are constructs that can be modified with interventions described by PP, more easily so than other relevant constructs on learning such as personality or social status. Therefore, this research is of interest for public education policies.

## Data Availability Statement

The datasets generated for this study are available on request to the corresponding author.

## Ethics Statement

The studies involving human participants were reviewed and approved by the Research Ethics Committee of the Federal University of Health Sciences of Porto Alegre (CAAE 52105315.7.0000.5345/opinion no. 1,625,066) and met all ethical criteria of human research. The patients/participants provided their written informed consent to participate in this study.

## Author Contributions

All authors listed have made a substantial, direct and intellectual contribution to the work, and approved it for publication.

## Conflict of Interest

The authors declare that the research was conducted in the absence of any commercial or financial relationships that could be construed as a potential conflict of interest.
